# Supplementary material for: The role of social determinants in COVID-19 hospitalization disparities by migration status in Stockholm, Sweden. A population-based cohort study
Source: Commun Med (Lond). 2026 Jan 15;6:93. doi: 10.1038/s43856-025-01357-w (PMC12886995; doi:10.1038/s43856-025-01357-w)
Supplement: Supplementary file 2 — Supplementary material [file 43856_2025_1357_MOESM2_ESM.docx]

**Supplementary Information**

Supplement to: The role of social determinants in COVID-19 hospitalization disparities by migration status in Stockholm, Sweden

**Content**

| **Supplementary 1. Fairlie decomposition** | |  |
| --- | --- | --- |
| **Table S1. List of countries/region within each regional groups. Regions such as “EU28 excluding the Nordic countries” appearing in the Total Population Register is because we have no access to the full list of countries but regions caused by the statistical secrecy.**  **Table S2. Swedish occupational codes included in essential occupational groups 1**  **Figure S1. Hospitalization prevalence for infectious & parasitic and respiratory system diagnosis before and during the COVID-19 pandemic.**  **Table S3. Descriptive statistics for the study population (Wave 2) from different country/region of birth. Except population and age, all values are in percentage.** | |  |
| **Figure S2. Hospitalization risks from a multivariable model stratified by country/region of origin group** | |  |
| **Table S4. Results from the decomposition analysis of the difference of COVID-19 hospitalization between the Swedish-born population and immigrant group by region of birth for Wave 1 of the pandemic. All percentage values are relative to the difference (row of ‘Difference’ in the table).** | |  |
| **Table S5. Results from the decomposition analysis of the difference of COVID-19 hospitalization between the Swedish-born population and immigrant group by region of birth for Wave 2 of the pandemic. All percentage values are relative to the difference (row of ‘Difference’ in the table).** | |  |
|  |  |  |
|  |  |  |
|  |  |  |
|  |  |  |
|  |  |  |

**Supplementary 1. Fairlie decomposition**

To systematically quantify the contributions of these factors, we use a statistical decomposition approach, Fairlie decomposition^2^, an extension of the Blinder-Oaxaca decomposition^3,4^ to nonlinear models. It partitions the difference into components – one part accounting for differential distribution of the measurable factors, and the other accounting for the remaining differences. Conceptually, our analysis considers a counterfactual scenario in which Swedish-born have the same distribution of risk factors as immigrants, thereby estimating how much of the hospitalization risk differences can be attributed to differential distributions of measured factors. Our analysis focuses on young adult immigrants residing in Stockholm Municipality during two pandemic waves (March 2020 to January 2021), clarifying the extent to which known differential distribution of social and health-related factors explain the differences and highlighting areas requiring further investigation.

Fairlie decomposition analysis usually starts with separate logistic regression models, in our case, for a Swedish-born individual and an individual from one immigrant group. The person’s probability of hospitalization will be estimated as follows:

| $\Pr(Y_{S}=1)=F\left( X^{S}\beta^{S} \right)= \frac{1}{1+e^{-X^{S}\beta^{S}}}$ | (1) |
| --- | --- |
| $\Pr(Y_{A}=1)=F\left( X^{A}\beta^{A} \right)= \frac{1}{1+e^{-X^{A}\beta^{A}}}$ | (2) |

where $Pr$ is the probability of hospitalization ($Y=1$), given coefficients $\beta$ for an individual with a vector of independent factors X. subscript S indicates the Swedish-born and subscript A indicates the immigrant.

Then the average predicted probability of hospitalization (hospitalization risk) for each group will be:

| $\bar{Y_{S}}=\frac{1}{N^{S}}\sum_{i=1}^{N^{S}} F\left( X_{i}^{S}\beta^{S} \right)$ | (3) |
| --- | --- |
| $\bar{Y_{A}}=\frac{1}{N^{A}}\sum_{i=1}^{N^{A}} F\left( X_{i}^{A}\beta^{A} \right)$ | (4) |

where $\bar{Y_{S}}$ and $\bar{Y_{A}}$ are hospitalization risks for the Swedish-born and the other immigrant group; $N^{S}$ and $N^{A}$ are the population size for each group.

The difference between Equation (3) and (4), which are the hospitalization risk difference between the two group, will then be decomposed into 2 components:

| 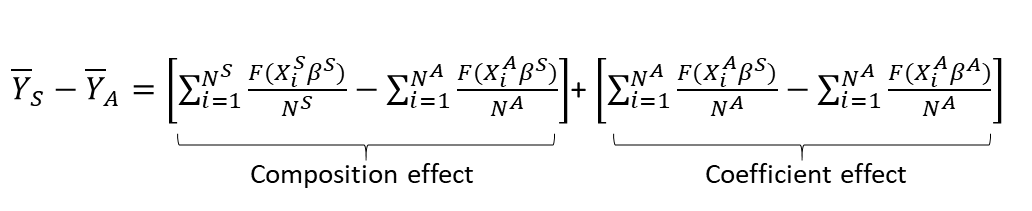 | (5) |
| --- | --- |

The first term (named *composition effect*) quantifies the proportion of hospitalization risk differences contributed from differential distribution of the measured factors. If this proportion is close to the actual risk differences, then hospitalization differences are primarily driven by differences in distribution of the factors. The second term (named *coefficient effect*) represents the remaining risk difference (i.e., unobserved factors or differential effects of explanatory factors). However, this component is sensitive to omitted factor bias and the contribution of each factor to coefficient effect cannot be quantified due to the nonlinear link function in the logistic regression^2^. Therefore, we focus primarily on the explained portion (composition effect) to address our research question. To still get some insight into coefficient effect, we ran logistic regression models for each group separately and presented the results in this Supplementary Information Figure S1.

In equation (5), coefficients from logistic regression of the Swedish-born are used to decompose the two effects. It is with the assumption that effects on factors are the same among all the immigrant groups as for the Swedish-born. However, to ensure a more robust estimate of coefficients, we used pooled coefficients (from logistic regressions including all study populations with dummy variables for regional groups) rather than relying solely on Swedish-born estimates. This approach is recommended for cases where the two comparison groups have substantially different sample sizes, which could otherwise lead to unstable estimates ^2,5^. In our case, it is also due to the unrobust estimates for coefficients of factor residential district, which is caused by the high clustering in residential area for different groups. In theory, if most pooled coefficients are close to the coefficients from Swedish-born estimates, the composition effects of the two will be similar. After applying pooled coefficients, equations (1) and (2) to predict a person’s probability of hospitalization ($Y=1$) change to the form:

| $\Pr(Y=1)=F\left( X\beta^{*}+\gamma D \right)$ |  | (6) |
| --- | --- | --- |

where $D$ is dummy for group (e.g., 0 = the Swedish-born as the reference group, 1 = one immigrant group); $\beta^{*}$ contains pooled coefficients (estimated from logistic regressions including all study populations, both Swedish-born and immigrants, with dummy variables for regional groups); $\gamma$ is coefficients for group dummy.

Then equation (5) changes to the form:

| 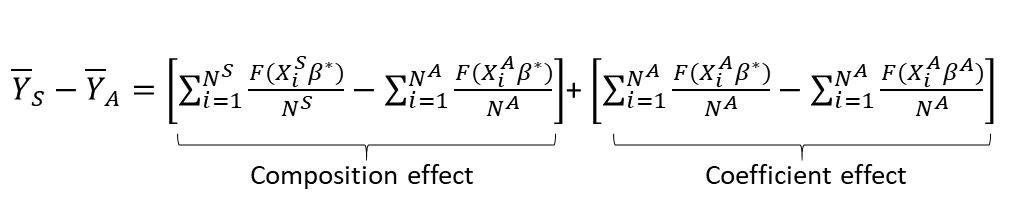 | (7) |
| --- | --- |

The total contribution from all factors (composition effect) for each pair of an immigrant group and the Swedish-born is calculated by substituting two sets factors to get predicted probabilities and take the difference between the average values of the two as shown in equation (7).

In order to calculate each independent factor’s contribution to the group difference in hospitalization risks for groups with different sample size, a random sample of the Swedish-born of equal size to another comparable immigrant group ($N^{A}$ in the case of $N^{A}< N^{S}$) was chosen, and the distribution of the immigrants replaced the Swedish-born distribution sequentially for each independent factor. For example, in the case of two independent factors, $X_{1}$ and $X_{2}$, the independent contribution of $X_{1}$ to the total risk difference can then be expressed as:

| $\frac{1}{N^{A}}\sum_{i,j=1}^{N^{A}} F\left( \beta_{0}^{*}+X_{1i}^{S}\beta_{1}^{*}+X_{2i}^{S}\beta_{2}^{*} \right)-F\left( \beta_{0}^{*}+X_{1j}^{A}\beta_{1}^{*}+X_{2i}^{S}\beta_{2}^{*} \right)$ | (8) |
| --- | --- |

Similarly, the contribution of $X_{2}$ can be expressed as:

| $\frac{1}{N^{A}}\sum_{i,j=1}^{N^{A}} F\left( \beta_{0}^{*}+X_{1j}^{A}\beta_{1}^{*}+X_{2i}^{S}\beta_{2}^{*} \right)-F\left( \beta_{0}^{*}+X_{1j}^{A}\beta_{1}^{*}+X_{2j}^{A}\beta_{2}^{*} \right)$ | (9) |
| --- | --- |

For each individual $j$ in the immigrant group, a corresponding individual $i$ from the Swedish-born is randomly selected in each iteration, and this resampling is repeated 1000 times. The formula above reflects a single iteration, with results averaged over all repetitions. The order of factors was randomized on each iteration to address the issue of path dependence where the contribution of each factor to the outcome gap can depend on the order in the models.

**Table S1. List of countries/region within each regional groups. Regions such as “EU28 excluding the Nordic countries” appearing in the Total Population Register is because we have no access to the full list of countries but regions caused by the statistical secrecy.**

| Regional groups | List of countries/region within each group |
| --- | --- |
| Africa | Somalia, Africa, Ethiopia, Eritrea |
| Middle East | Iran, Middle East, Iraq, Syria, Turkey, Lebanon, Afghanistan |
| Other Nordic Countries | Finland, Denmark, Norway, Nordic countries except Sweden |
| Other European Countries | Germany, Italy, Estonia, Poland, Austria, Czechoslovakia, EU28 excluding the Nordic countries, Greece, France, Hungary, Russia, Romania, Europe excluding EU28 and the Nordic countries, Yugoslavia, Soviet Union, Croatia, Spain, Netherlands |
| Rest of the World | South America, China, Chile, North America, Asia, Philippines, Oceania, Thailand, Colombia, Pakistan, Vietnam, India, Peru, Brazil, Sri Lanka, Stateless |

Note: Regional groups reflect the categories we have obtained from Statistics Sweden. For some individuals, country of birth is recorded at the national level (e.g., Somalia), whereas for others only a broader regional or continental code is available (e.g., “Africa” or “Middle East”). These broader categories arise due to statistical secrecy and disclosure control procedures. Our groupings therefore directly follow the register definitions without alteration.

**Table S2. Swedish occupational codes included in essential occupational groups** ^1^

| Essential Occupations | SSYK 2012 |
| --- | --- |
| Care workers | 221, 222, 223, 532, 533, 534; excluding 2222, 2225, 2233, 2234 |
| Taxi- and bus | 8321, 8331 |
| Meat packers | 7611 |
| Teachers | 234, 5311 |
| Service sector | 522, 941, 523 |
| Policemen, security guards | 5412, 5413, 3360 |
| Postal workers, delivery | 4420, 8329 |
| Cleaners | 9111 |

**Figure S1. Hospitalization prevalence for infectious & parasitic and respiratory system** **diagnosis before and during the COVID-19 pandemic.**


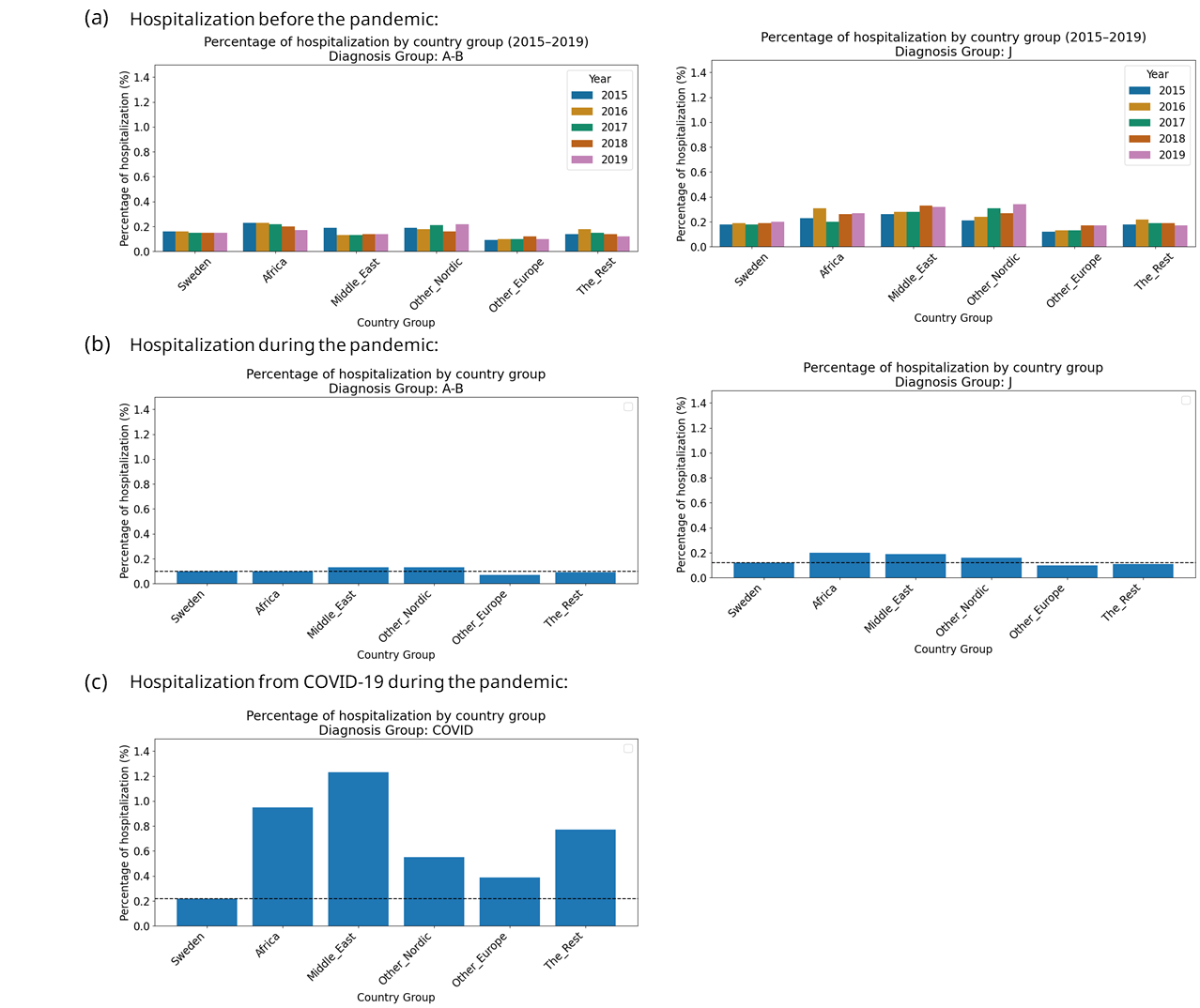


Legend: Hospitalization prevalence for infectious & parasitic diagnosis (ICD -10: A00-B99) and respiratory system diagnosis (ICD-10: J00-J99) for each year (a) before the pandemic and (b) during the pandemic compared with (c) hospitalization risk from COVID-19 during the pandemic.

**Table S3. Descriptive statistics for the study population (Wave 2) from different country/region of birth. Except population and age, all values are in percentage.**

| Factors | All Population | Sweden | Africa | Middle East | Other Nordic Countries | Other European Countries | Rest of the World |
| --- | --- | --- | --- | --- | --- | --- | --- |
| Population | 577 537 | 437 953 | 24 423 | 36 192 | 10 198 | 34 572 | 34 199 |
| Person-time, total amount (average) | 240 210  (0.4159) | 182 188  (0.4160) | 10 154  (0.4158) | 15 034  (0.4154) | 4 240  (0.4158) | 14 378  (0.4159) | 14 216  (0.4157) |
| Percentage of Total Population | | 75.8 | 4.2 | 6.3 | 1.8 | 6.0 | 5.9 |
| Sex |  |  |  |  |  |  |  |
| Female | 50.4 | 50.1 | 48.8 | 46.5 | 61.8 | 52.2 | 54.3 |
| Male | 49.6 | 49.9 | 51.2 | 53.5 | 38.2 | 47.8 | 45.7 |
| Age |  |  |  |  |  |  |  |
| Mean | 41.0 | 40.2 | 42.4 | 44.2 | 49.1 | 43.0 | 42.2 |
| SD | 12.9 | 13.1 | 11.6 | 12.0 | 12.2 | 11.6 | 11.3 |
| Pre-existing Medical Conditions | |  |  |  |  |  |  |
| No | 90.7 | 91.1 | 88.5 | 87.9 | 85.2 | 92.3 | 90.6 |
| Yes | 9.3 | 9.0 | 11.5 | 12.1 | 14.9 | 7.8 | 9.5 |
| Occupation Type |  |  |  |  |  |  |  |
| Essential | 20.2 | 17.1 | 44.9 | 30.8 | 17.9 | 21.0 | 30.1 |
| Non-essential | 57.7 | 63.2 | 24.1 | 34.4 | 62.3 | 50.7 | 42.4 |
| Unemployed or Unregistered | 22.1 | 19.7 | 31.0 | 34.9 | 19.8 | 28.3 | 27.5 |
| Income Level |  |  |  |  |  |  |  |
| Low | 33.3 | 30.0 | 48.7 | 47.3 | 27.5 | 40.6 | 44.1 |
| Medium | 33.3 | 32.6 | 39.3 | 35.2 | 33.8 | 33.5 | 36.7 |
| High | 33.3 | 37.4 | 12.0 | 17.5 | 38.7 | 25.9 | 19.3 |
| Education Level |  |  |  |  |  |  |  |
| Primary | 10.9 | 8.9 | 27.4 | 23.0 | 7.4 | 8.5 | 15.3 |
| Upper secondary | 31.7 | 31.5 | 42.2 | 34.8 | 28.9 | 26.0 | 30.0 |
| Post secondary | 56.0 | 59.0 | 27.2 | 40.3 | 61.1 | 57.0 | 51.3 |
| Missing | 1.4 | 0.5 | 3.2 | 1.9 | 2.6 | 8.7 | 3.4 |
| District |  |  |  |  |  |  |  |
| Bromma | 5.5 | 5.9 | 2.3 | 3.5 | 6.0 | 4.9 | 4.8 |
| Brännkyrka | 5.5 | 5.6 | 4.0 | 5.0 | 5.0 | 5.3 | 6.0 |
| Enskede | 4.6 | 5.1 | 2.4 | 2.2 | 4.5 | 3.2 | 3.8 |
| Essinge | 1.0 | 1.2 | 0.2 | 0.3 | 1.3 | 0.9 | 0.7 |
| Farsta | 6.0 | 5.9 | 6.6 | 5.4 | 5.3 | 6.2 | 8.1 |
| Hägersten | 8.1 | 9.0 | 2.7 | 3.9 | 8.4 | 7.2 | 6.1 |
| Hässelby | 3.8 | 3.1 | 7.1 | 7.4 | 4.0 | 5.4 | 4.8 |
| Högalid | 3.4 | 3.9 | 0.7 | 1.0 | 3.8 | 2.4 | 2.1 |
| Kista | 3.3 | 1.5 | 12.5 | 13.3 | 2.6 | 4.4 | 7.4 |
| Kungsholmen | 2.1 | 2.4 | 0.4 | 0.7 | 2.3 | 1.5 | 1.2 |
| Skarpnäck | 5.2 | 5.5 | 3.8 | 2.6 | 5.7 | 5.0 | 4.9 |
| Skärholmen | 3.6 | 2.0 | 9.0 | 12.9 | 2.1 | 7.1 | 7.3 |
| Spånga | 5.3 | 3.1 | 27.5 | 15.0 | 3.9 | 6.2 | 6.6 |
| Stockholms Adolf Fredrik | 1.0 | 1.1 | 0.2 | 0.5 | 1.0 | 0.9 | 0.6 |
| Stockholms Engelbrekt | 3.0 | 3.2 | 0.7 | 1.3 | 3.4 | 3.7 | 2.7 |
| Stockholms Gustav Vasa | 1.6 | 1.8 | 0.3 | 0.6 | 1.7 | 1.4 | 0.9 |
| Stockholms Hedvig Eleonora | 1.1 | 1.3 | 0.2 | 0.4 | 1.2 | 0.9 | 0.5 |
| Stockholms Katarina | 3.9 | 4.5 | 1.2 | 1.3 | 4.3 | 2.7 | 2.5 |
| Stockholms Maria Magdalena | 2.1 | 2.4 | 0.5 | 0.6 | 2.4 | 1.5 | 1.4 |
| Stockholms Oscar | 3.9 | 4.5 | 0.5 | 1.2 | 4.0 | 2.9 | 2.1 |
| Stockholms Sankt Göran | 4.7 | 5.2 | 1.1 | 2.7 | 5.3 | 3.9 | 3.4 |
| Stockholms Sankt Johannes | 1.3 | 1.4 | 0.3 | 0.6 | 1.2 | 1.3 | 0.9 |
| Stockholms Sankt Matteus | 3.5 | 4.0 | 0.5 | 1.2 | 3.6 | 2.8 | 2.1 |
| Stockholms Sofia | 4.2 | 4.7 | 0.7 | 1.4 | 4.9 | 3.3 | 2.7 |
| Stockholms domkyrkodistrikt | 0.5 | 0.6 | 0.1 | 0.3 | 0.6 | 0.6 | 0.4 |
| Vantör | 5.5 | 4.7 | 8.6 | 6.5 | 4.7 | 8.3 | 9.7 |
| Vällingby | 3.7 | 3.2 | 5.7 | 7.6 | 3.6 | 4.4 | 5.1 |
| Västerled | 2.9 | 3.4 | 0.5 | 0.7 | 3.3 | 2.1 | 1.3 |

**Figure S2. Hospitalization risks from a multivariable model stratified by country/region of origin group**

**
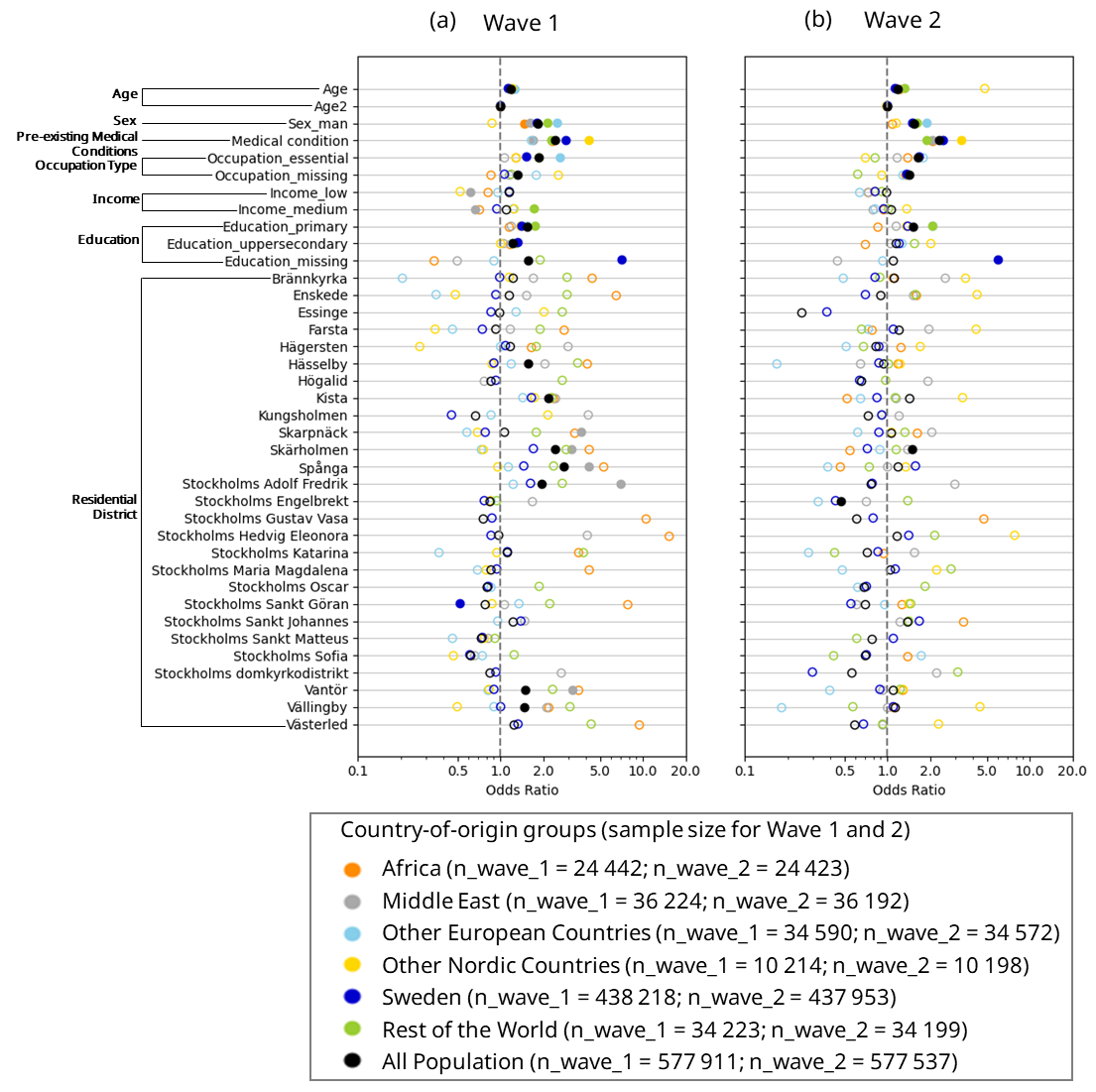
**

Legend: Odds ratios from multivariable logistic regression for each country-of-origin group and entire population for (a) Wave 1, and (b) Wave 2. Odds ratios with a p-value less than 0.05 are marked with filled circle, those with a p-value greater than 0.05 are marked with empty circles. The sample sizes for each country-of-origin group and entire population are marked in the legend. All tests were two-sided. No adjustment was made for multiple comparisons.

**Table S4. Results from the decomposition analysis of the difference of COVID-19 hospitalization between the Swedish-born population and immigrant group by region of birth for Wave 1 of the pandemic. All percentage values are relative to the difference (row of ‘Difference’ in the table).**

|  | Africa | | Middle East | | Other Nordic Countries | | Other European Countries | | Rest of the World | |
| --- | --- | --- | --- | --- | --- | --- | --- | --- | --- | --- |
|  | Coefficient (95%CI) | % of Difference Explained | Coefficient (95%CI) | % of Difference Explained | Coefficient (95%CI) | % of Difference Explained | Coefficient (95%CI) | % of Difference Explained | Coefficient (95%CI) | % of Difference Explained |
| Risk of the Swedish origin | 0.00136 |  | 0.00136 |  | 0.00136 |  | 0.00136 |  | 0.00136 |  |
| Risk of immigrant groups | 0.00679 |  | 0.00787 |  | 0.00323 |  | 0.00214 |  | 0.00491 |  |
| Difference | -0.00543 |  | -0.00650 |  | -0.00187 |  | -0.00077 |  | -0.00354 |  |
| Composition effect | -0.00112 |  | -0.00114 |  | -0.00082 |  | -0.00037 |  | -0.00043 |  |
| Composition effect (%) | 20.61% |  | 17.56% |  | 43.77% |  | 47.68% |  | 12.00% |  |
| Coefficient effect (%) | 79.39% |  | 82.44% |  | 56.23% |  | 52.32% |  | 88.00% |  |
| Contribution of different factors | | | | | | | | | | |
| Age | -0.00008 | 1.56% ** | -0.00027 | 4.10% *** | -0.00061 | 32.71% *** | -0.00013 | 16.19% *** | -0.00007 | 1.89% *** |
|  | (-0.00013, -0.00004) |  | (-0.00031, -0.00023) |  | (-0.00070, -0.00053) |  | (-0.00016, -0.00009) |  | (-0.00010, -0.00003) |  |
| Sex | 0.00000 | 0.00% | -0.00002 | 0.33% | 0.00014 | -7.23% *** | 0.00003 | -4.22% ** | 0.00005 | -1.53% *** |
|  | (-0.00003, 0.00003) |  | (-0.00005, 0.00000) |  | (0.00009, 0.00018) |  | (0.00001, 0.00006) |  | (0.00003, 0.00008) |  |
| Pre-existing medical conditions | -0.00008 | 1.42% *** | -0.00013 | 1.94% *** | -0.00021 | 11.02% *** | 0.00003 | -4.36% ** | -0.00001 | 0.35% |
|  | (-0.00010, -0.00005) |  | (-0.00016, -0.00009) |  | (-0.00025, -0.00016) |  | (0.00001, 0.00006) |  | (-0.00003, 0.00001) |  |
| Occupation type | -0.00029 | 5.40% *** | -0.00019 | 2.90% *** | -0.00004 | 2.37% ** | -0.00008 | 10.80% *** | -0.00016 | 4.49% *** |
|  | (-0.00041, -0.00017) |  | (-0.00029, -0.00009) |  | (-0.00008, -0.00001) |  | (-0.00013, -0.00004) |  | (-0.00023, -0.00009) |  |
| Income | 0.00002 | -0.43% | 0.00003 | -0.40% | 0.00001 | -0.45% | 0.00001 | -1.78% | 0.00002 | -0.47% |
|  | (-0.00008, 0.00012) |  | (-0.00008, 0.00013) |  | (-0.00003, 0.00004) |  | (-0.00004, 0.00007) |  | (-0.00005, 0.00009) |  |
| Education | -0.00018 | 3.28% *** | -0.00014 | 2.14% *** | -0.00004 | 2.13% ** | -0.00007 | 9.11% | -0.00007 | 1.86% ** |
|  | (-0.00027, -0.00009) |  | (-0.00022, -0.00006) |  | (-0.00006, -0.00002) |  | (-0.00015, 0.00001) |  | (-0.00011, -0.00003) |  |
| District | -0.00051 | 9.37% *** | -0.00043 | 6.56% *** | -0.00006 | 3.17% ** | -0.00017 | 21.99% *** | -0.00019 | 5.44% *** |
|  | (-0.00071, -0.00031) |  | (-0.00060, -0.00025) |  | (-0.00010, -0.00002) |  | (-0.00024, -0.00010) |  | (-0.00028, -0.00010) |  |

*p<0.05; **p<0.01; ***p<0.001.

The decomposition analysis was based on two-sided tests of the underlying logistic regression coefficients. The significance of the decomposition components was assessed using bootstrapped standard errors (two-sided).

**Table S5. Results from the decomposition analysis of the difference of COVID-19 hospitalization between the Swedish born population and immigrant group by region of birth for Wave 2 of the pandemic. All percentage values are relative to the difference (row of ‘Difference’ in the table).**

|  | Africa | | Middle East | | Other Nordic Countries | | Other European Countries | | Rest of the World | |
| --- | --- | --- | --- | --- | --- | --- | --- | --- | --- | --- |
|  | Coefficient (95%CI) | % of Difference Explained | Coefficient (95%CI) | % of Difference Explained | Coefficient (95%CI) | % of Difference Explained | Coefficient (95%CI) | % of Difference Explained | Coefficient (95%CI) | % of Difference Explained |
| Risk of the Swedish origin | 0.00095 |  | 0.00095 |  | 0.00095 |  | 0.00095 |  | 0.00095 |  |
| Risk of immigrant groups | 0.00295 |  | 0.00478 |  | 0.00255 |  | 0.00179 |  | 0.00295 |  |
| Difference | -0.00200 |  | -0.00383 |  | -0.00160 |  | -0.00084 |  | -0.00200 |  |
| Composition effect | -0.00031 |  | -0.00043 |  | -0.00062 |  | -0.00011 |  | -0.00014 |  |
| Composition effect (%) | 15.69% |  | 11.27% |  | 38.58% |  | 12.75% |  | 6.89% |  |
| Coefficient effect (%) | 84.31% |  | 88.73% |  | 61.42% |  | 87.25% |  | 93.11% |  |
| Contribution of different factors | | | | | | | | | | |
| Age | -0.00005 | 2.69% ** | -0.00019 | 4.97% *** | -0.00049 | 30.93% *** | -0.00010 | 11.30% *** | -0.00004 | 1.98% ** |
|  | (-0.00008, -0.00002) |  | (-0.00022, -0.00016) |  | (-0.00057, -0.00042) |  | (-0.00012, -0.00007) |  | (-0.00007, -0.00001) |  |
| Sex | -0.00001 | 0.31% | -0.00002 | 0.51% ** | 0.00007 | -4.07% *** | 0.00001 | -1.60% | 0.00002 | -1.17% ** |
|  | (-0.00002, 0.00001) |  | (-0.00003, -0.00001) |  | (0.00003, 0.00010) |  | (0.00000, 0.00003) |  | (0.00001, 0.00004) |  |
| Pre-existing medical Conditions | -0.00006 | 3.09% *** | -0.00009 | 2.30% *** | -0.00015 | 9.45% *** | 0.00001 | -1.62% * | -0.00001 | 0.73% |
|  | (-0.00008, -0.00004) |  | (-0.00011, -0.00006) |  | (-0.00019, -0.00011) |  | (0.00000, 0.00003) |  | (-0.00003, 0.00000) |  |
| Occupation type | -0.00014 | 7.16% ** | -0.00012 | 3.05% ** | -0.00004 | 2.48% ** | -0.00006 | 6.54% ** | -0.00009 | 4.37% ** |
|  | (-0.00023, -0.00006) |  | (-0.00019, -0.00004) |  | (-0.00007, -0.00001) |  | (-0.00009, -0.00002) |  | (-0.00014, -0.00003) |  |
| Income | 0.00006 | -3.04% | 0.00008 | -1.96% | 0.00003 | -1.86% | 0.00004 | -4.75% | 0.00005 | -2.41% |
|  | (-0.00001, 0.00013) |  | (0.00000, 0.00015) |  | (-0.00001, 0.00006) |  | (0.00000, 0.00008) |  | (-0.00001, 0.00010) |  |
| Education | -0.00009 | 4.60% ** | -0.00008 | 2.03% ** | -0.00002 | 1.37% * | -0.00001 | 0.81% | -0.00003 | 1.66% * |
|  | (-0.00016, -0.00003) |  | (-0.00013, -0.00002) |  | (-0.00004, 0.00000) |  | (-0.00005, 0.00003) |  | (-0.00006, 0.00000) |  |
| District | -0.00002 | 0.89% | -0.00001 | 0.38% | 0.00000 | 0.27% | -0.00002 | 2.00% | -0.00003 | 1.74% |
|  | (-0.00013, 0.00009) |  | (-0.00012, 0.00009) |  | (-0.00003, 0.00002) |  | (-0.00006, 0.00003) |  | (-0.00009, 0.00002) |  |

*p<0.05; **p<0.01; ***p<0.001.

The decomposition analysis was based on two-sided tests of the underlying logistic regression coefficients. The significance of the decomposition components was assessed using bootstrapped standard errors (two-sided).

**References:**

1. Billingsley, S. *et al.* COVID-19 mortality across occupations and secondary risks for elderly individuals in the household: A population register-based study. *Scand. J. Work. Environ. Health* **48**, 52–60 (2022).

2. Fairlie, R. W. An extension of the Blinder-Oaxaca decomposition technique to logit and probit models. *J. Econ. Soc. Meas.* **30**, 305–316 (2005).

3. Blinder, A. S. Wage Discrimination: Reduced Form and Structural Estimates. *J. Hum. Resour.* **8**, 436–455 (1973).

4. Oaxaca, R. Male-Female Wage Differentials in Urban Labor Markets. *Int. Econ. Rev.* **14**, 693–709 (1973).

5. Rahimi, E. & Hashemi Nazari, S. S. A detailed explanation and graphical representation of the Blinder-Oaxaca decomposition method with its application in health inequalities. *Emerg. Themes Epidemiol.* **18**, 12 (2021).
