# Supplementary material for: The role of social determinants in COVID-19 hospitalization disparities by migration status in Stockholm, Sweden. A population-based cohort study
Source: Commun Med (Lond). 2026 Jan 15;6:93. doi: 10.1038/s43856-025-01357-w (PMC12886995; doi:10.1038/s43856-025-01357-w)
Supplement: Supplementary file 3 — Description of Additional Supplementary Files [file 43856_2025_1357_MOESM3_ESM.pdf]

Description of Additional Supplementary Files

File name: Supplementary Data 1-2

Description: Source data behind the graphs in the paper
